# Supplementary material for: Intracellular pH dynamics regulates intestinal stem cell lineage specification
Source: Nat Commun. 2023 Jun 23;14:3745. doi: 10.1038/s41467-023-39312-9 (PMC10290085; doi:10.1038/s41467-023-39312-9)
Supplement: Supplementary file 1 — Supplementary Information [file 41467_2023_39312_MOESM1_ESM.pdf]

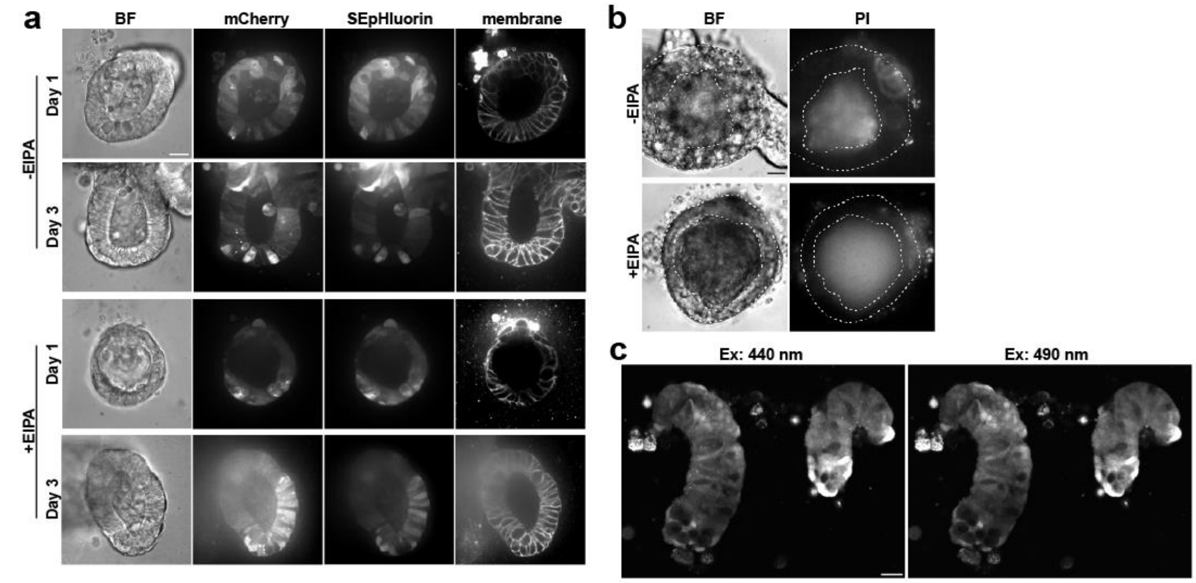

Supplementary Fig.1: Microscopy for pHi and cell viability in organoids.

a, Representative live-cell images of mCherry-SEpHluorin in the crypt region in day 1 and day 3 organoids in the absence (Control) and presence of 5 μM EIPA. Images are selected from n=3 biologically independent experiments. Panels show brightfield (BF) individual and merged mCherry and SEpHluorin signal, and far-red membrane dye to determine individual cells. **b,** Propidium iodide (PI) staining as an index of cell viability in day 3 organoids (n = 3 biologically independent experiments) in the absence and presence of EIPA. Space between dashed circles indicates epithelial layer. Inner dashed circle indicates the lumen containing apoptotic cells with positive staining. **c,** Representative live-cell images of BCECF loaded freshly isolated crypts (n=2 biologically independent experiments). Panels show pH insensitive (Ex: 440 nm) and pH sensitive (Ex: 490 nm) channels. Ex, excitation wavelength. All scale bars, 20 μm.

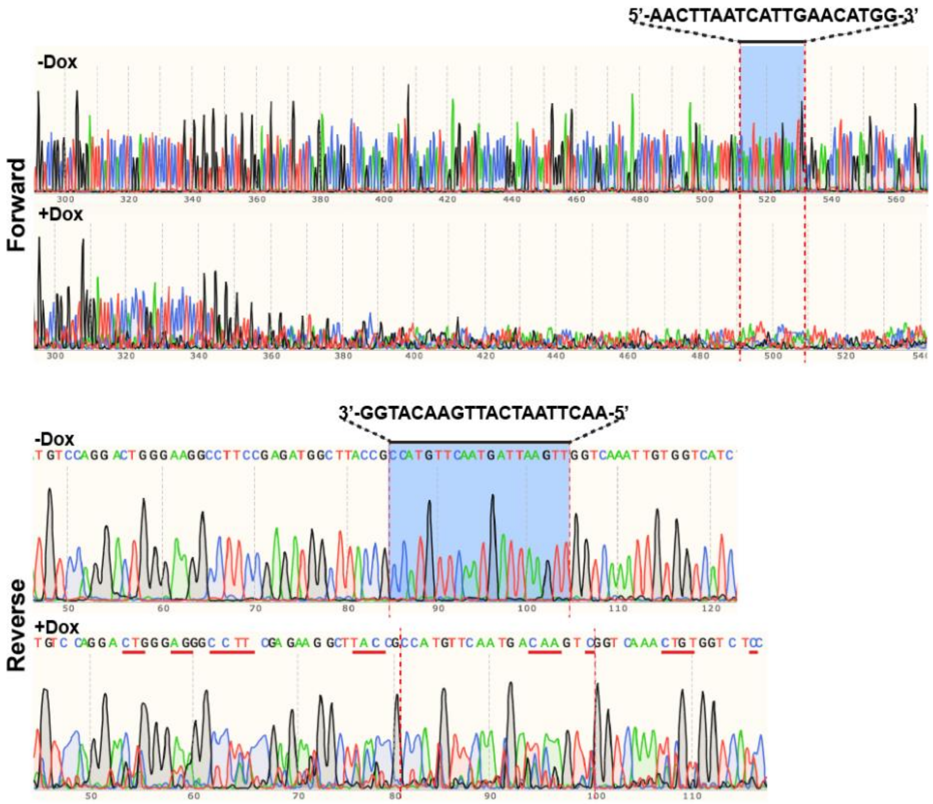

Supplementary Fig.2: NHE1 CRSIPR-Cas9 silencing in organoids.

Validation of NHE1 silencing. Results are generated using forward and reverse sequencing primers, respectively. The Cas9 guide RNA targeting region is indicated by a black line with red dashed lines. Reduction of signal in forwarding sequencing indicates large disruption of DNA sequence. Horizontal red solid lines in reverse sequencing indicate CRISPR indels.

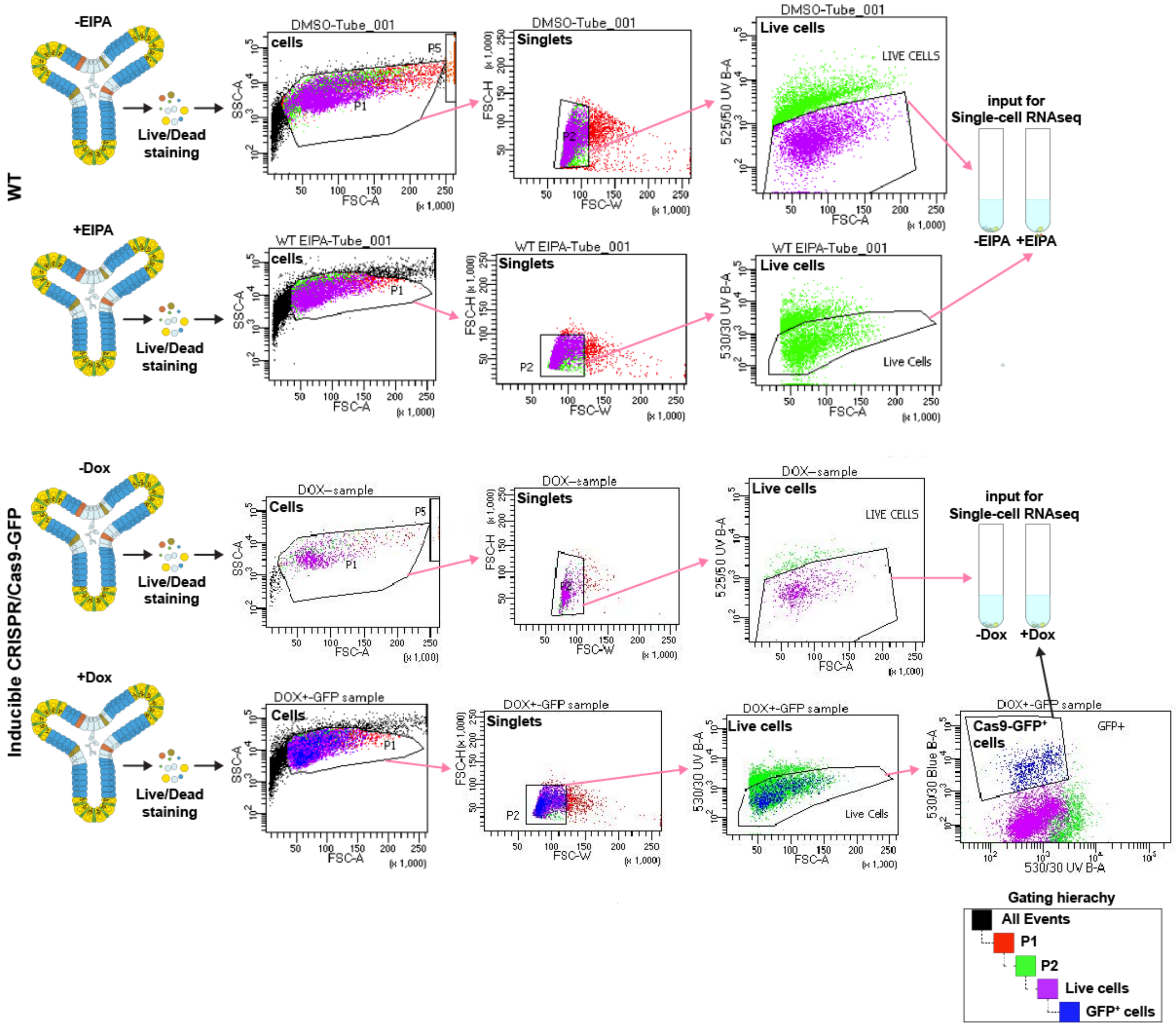

Supplementary Fig.3: FACS for single-cell RNA sequencing.

Schematic and flow cytometry plots show fluorescence-activated cell sorting (FACS) gating strategies for single-cell RNA sequencing. In brief, the organoids are dissociated into individual cells, stained by Live/Dead (Invitrogen, L23105, 1:1000), and then loaded onto FACS to obtain the desired type of single live cells before single-cell RNA sequencing. Top panels, FACS gating strategy for WT (-/+EIPA) organoids. Bottom panels, FACS gating strategy for doxycycline (Dox) inducible- NHE1 CRISPR/Cas9-GFP (-/+Dox) organoids.

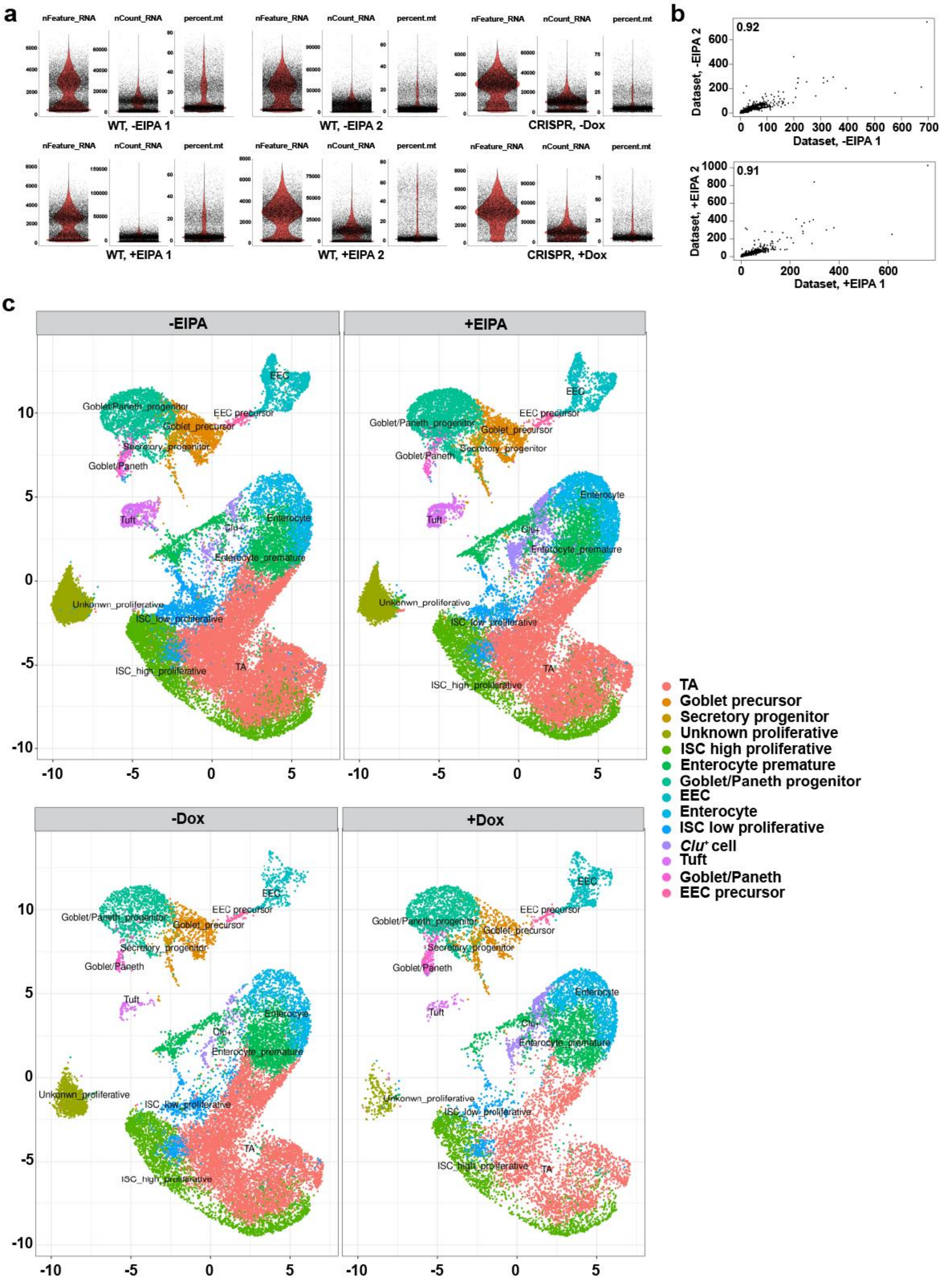

Supplementary Fig.4: Quality control of single-cell RNA sequencing.

a, Visualization of the quality control metrics of datasets (Methods). Violin plots showing nFeature_RNA (unique genes), nCount_RNA (total number of RNAs), and percent.Mt (mitochondrial RNA, low quality and dying cells). **b,** Scatter plots showing the relationship between biological replicates of organoids preparation (Methods). The Pearson correlation coefficient, indicated in the upper left of each graph, is calculated using the average expression profiles of individual datasets. **c,** UMAP visualization of cell-identity clusters sorted by the conditions, WT+/-EIPA, CRISPR+/-Dox.

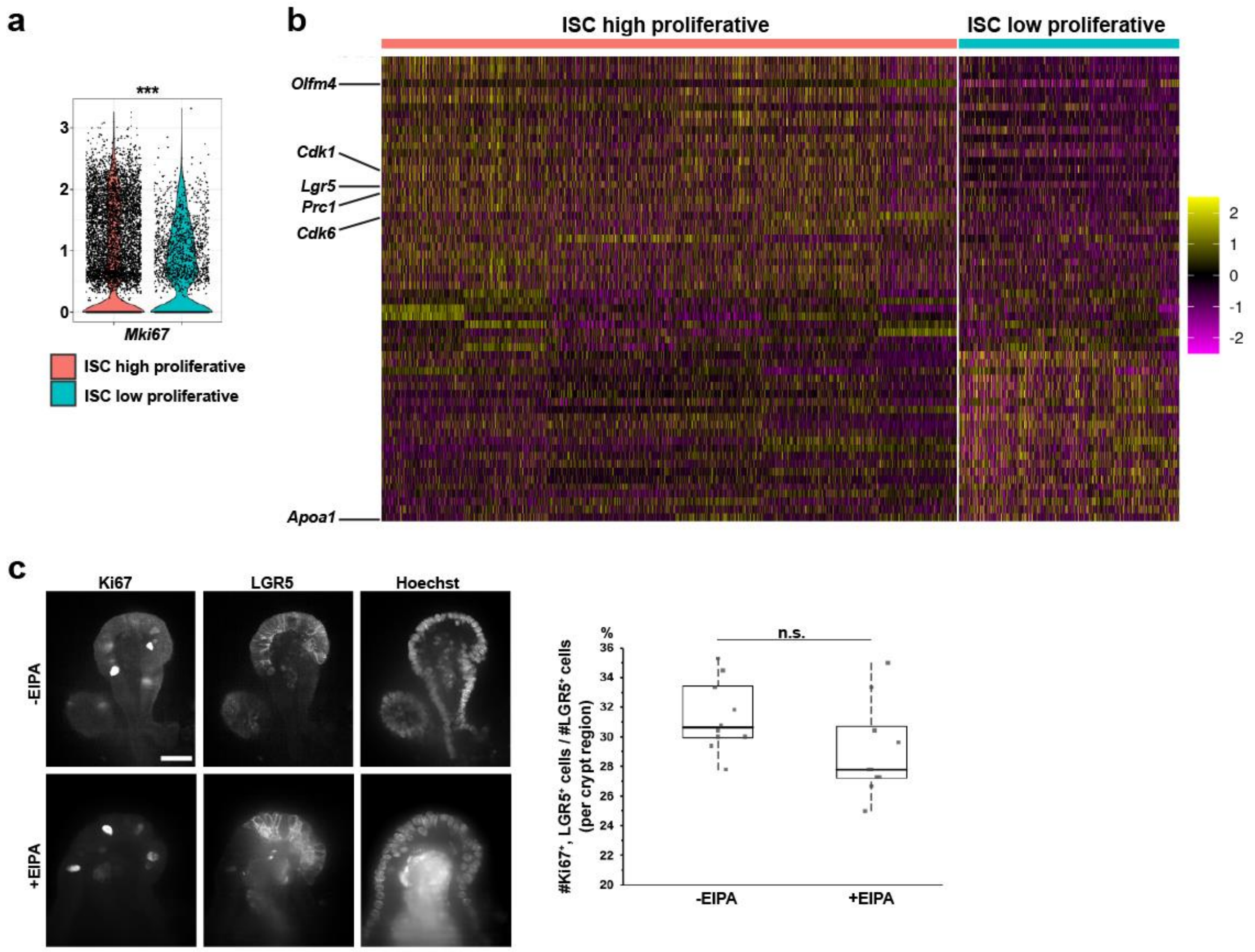

Supplementary Fig.5: ISC subtypes and proliferation.

a, Violin plot showing the expression level of *Mki67* between the distinct ISC subclusters. Data are assessed using the two-sided Student's t-test. ***$p < 0.001$, p = 4.54E-19. **b,** Heatmap showing signatures of high proliferative and low proliferative ISC subtypes. Colored expression level is relative to the mean expression of cell population, 0 (population mean) ± 2 (SD). Cell cycle signature genes (*Cdk1*, *Prc1*, and *Cdk6*), Stem cell-specific genes (*Lgr5* and *Olfm4*) and the absorptive-associated gene (*Apoa1*) are highlighted (left row). **c,** Ki67 immunolabeling of day 3 *Lgr5*$^{\text{DTR-GFP}}$ organoids maintained in the absence (Control) or presence of EIPA. Left panel representative images, right panel quantified data from crypt regions in organoid preparations (n=1 biologically independent experiment, two-sided Tukey-Kramer test) indicating no difference (n.s, no statistically significant difference, p =0.08) in the number of Ki67$^+$, LGR5$^+$ cells per the number of LGR5$^+$ cells between control and EIPA conditions. Box plots are minimum to maximum, the box shows 25th-75th percentiles, and the central line is the median. Source data are provided as a Source Data file. Scale bar, 20 μm.

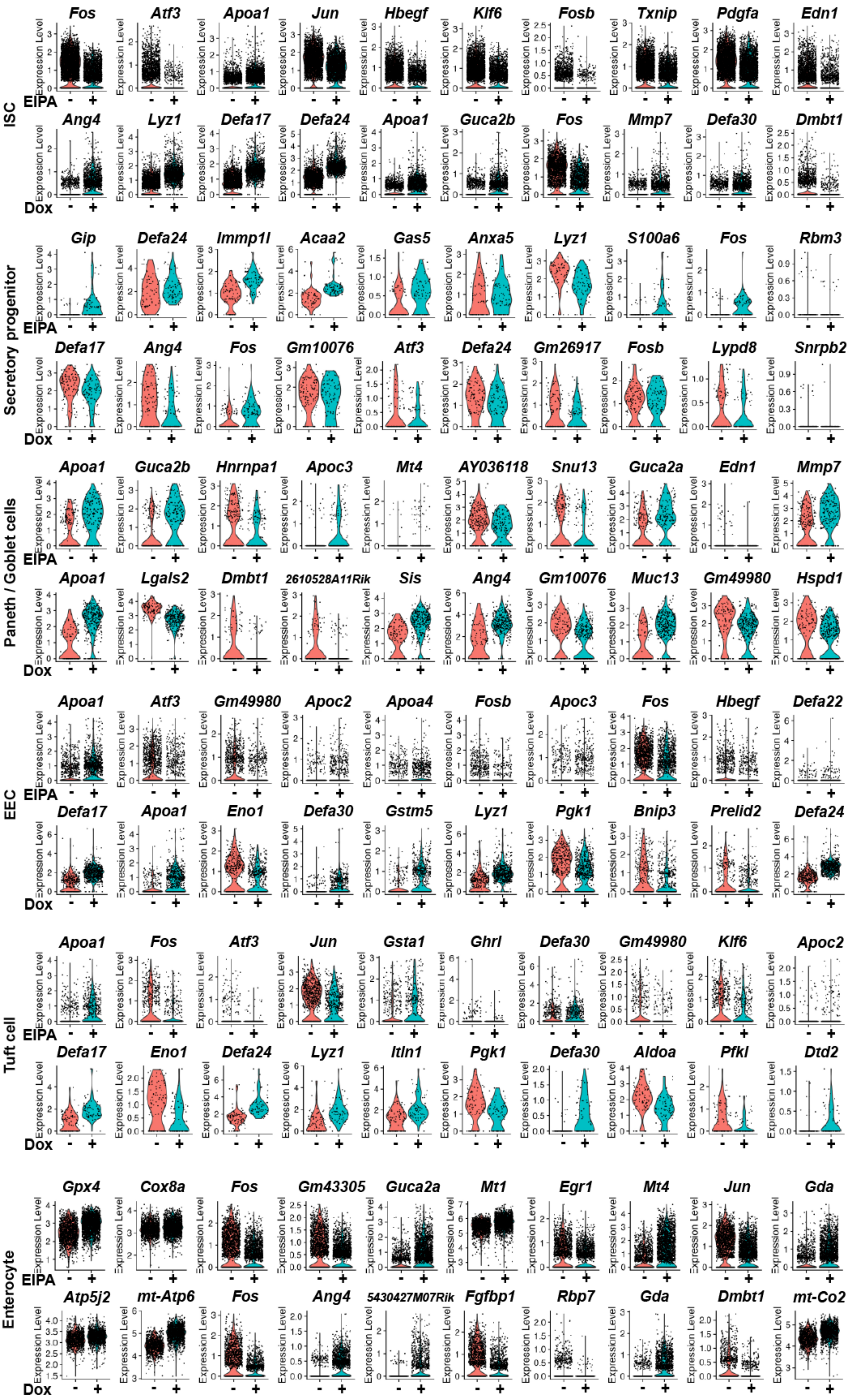

ISC
EIPA
Fos
Atf3
Apoa1
Jun
Hbegf
Klf6
Fosb
Txnip
Pdgfa
Edn1
Dox
Ang4
Lyz1
Defa17
Defa24
Apoa1
Guca2b
Fos
Mmp7
Defa30
Dmbt1
Secretory progenitor
EIPA
Gip
Defa24
Immp1l
Acaa2
Gas5
Anxa5
Lyz1
S100a6
Fos
Rbm3
Dox
Defa17
Ang4
Fos
Gm10076
Atf3
Defa24
Gm26917
Fosb
Lypd8
Snrpb2
Paneth / Goblet cells
EIPA
Apoa1
Guca2b
Hnrnpa1
Apoc3
Mt4
AY036118
Snu13
Guca2a
Edn1
Mmp7
Dox
Apoa1
Lgals2
Dmbt1
2610528A11Rik
Sis
Ang4
Gm10076
Muc13
Gm49980
Hspd1
EEC
EIPA
Apoa1
Atf3
Gm49980
Apoc2
Apoa4
Fosb
Apoc3
Fos
Hbegf
Defa22
Dox
Defa17
Apoa1
Eno1
Defa30
Gstm5
Lyz1
Pgk1
Bnip3
Prelid2
Defa24
Tuft cell
EIPA
Apoa1
Fos
Atf3
Jun
Gsta1
Ghrl
Defa30
Gm49980
Klf6
Apoc2
Dox
Defa17
Eno1
Defa24
Lyz1
Itln1
Pgk1
Defa30
Aldoa
Pfkl
Dtd2
Enterocyte
EIPA
Gpx4
Cox8a
Fos
Gm43305
Guca2a
Mt1
Egr1
Mt4
Jun
Gda
Dox
Atp5j2
mt-Atp6
Fos
Ang4
5430427M07Rik
Fgfbp1
Rbp7
Gda
Dmbt1
mt-Co2
Expression Level
-
+

Supplementary Fig.6: Differential gene expression per cell cluster.
Violin plots show the top 10 changes in gene expression ($p < 0.001$) in main cell clusters upon NHE1 inhibition via EIPA or CRISPR (+Dox). Statistical analysis by two-sided DECENT test.

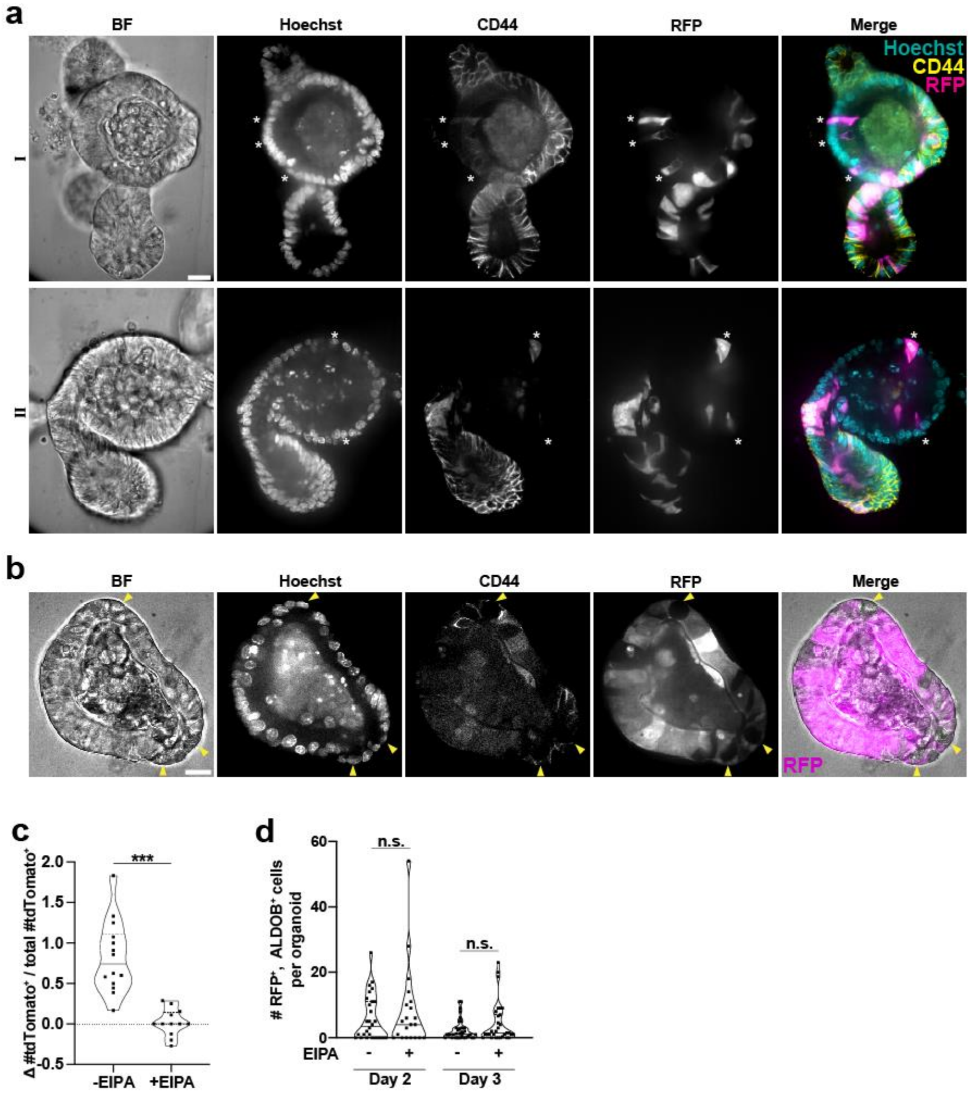

Supplementary Fig.7: Lineage tracing of crypt and villus cells in *Lgr5*^{CreER};*Rosa26*^{RFP} organoids.

a, Representative images show the presence of labeled *Lgr5*$^{+}$ ISC progeny (RFP^{+}) in crypt (CD44^{+}) and villus (CD44^{-}) regions of day 3 organoids. Organoids are treated with 4-hydroxytamoxifen on day 1 followed by washing and reseeding on day 2. Immunolabeled images show 2 examples (I, II) of day 3 reseeded organoids from n=6 biologically independent experiments. **b,** Example of an EIPA-treated organoid with robust labeling in the *Lgr5*$^{+}$ ISC progeny from n=3 biologically independent experiments. **c,** Quantification of lineage tracing for secretory cells in *Atoh1*$^{\text{CreERT2}}$;*Rosa26*^{tdTomato} organoids (see Fig.5f). Data are normalized to all labelled secretory cells (tdTomato^{+}) seen on day 2 (n=3 biologically independent experiments, two-sided Welch's t-test, p = 7.841E-06). Source data are provided as a Source Data file. **d,** Quantification of the number of RFP^{+}, ALDOB^{+} double-labeled cells in the villus region (CD44^{-}) in day 2 and day 3 organoids maintained in the absence (control) and presence of EIPA (n=4 biologically independent experiments, two-sided Mann-Whitney test, n.s., not statistically significant, $^{\text{Day 2}}p$ = 0.8583; $^{\text{Day 3}}p$ = 0.3579). Arrowhead indicates Paneth cells, which are identified by visible dense granules and large cell size. Source data are provided as a Source Data file. All scale bars, 20 μm.

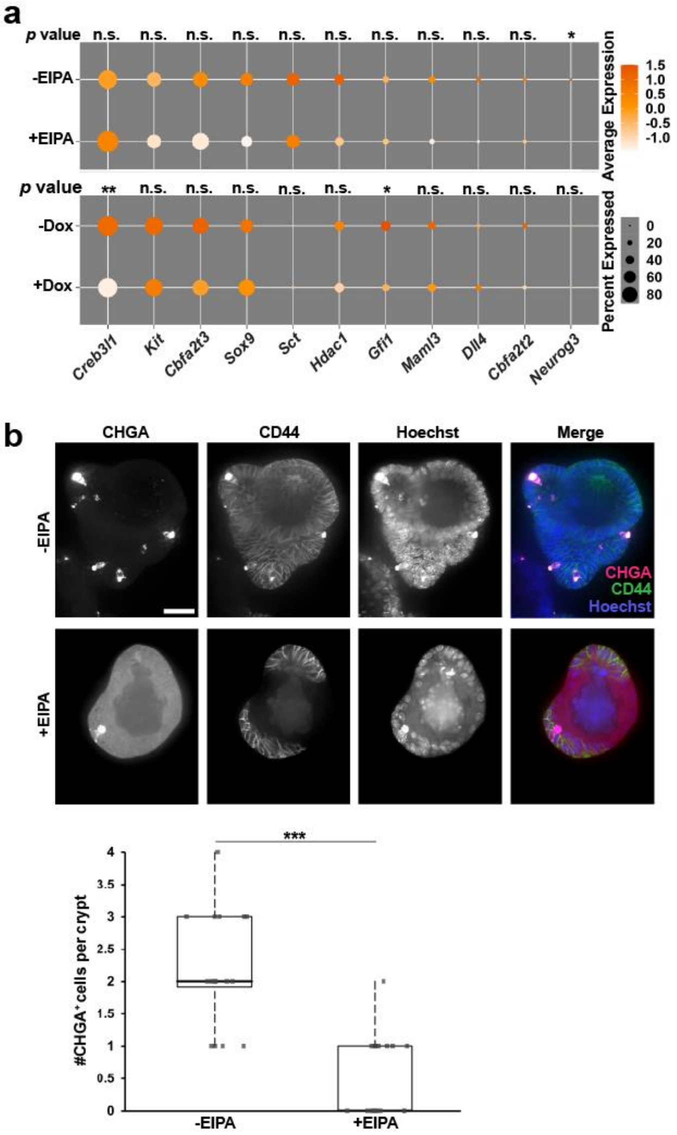

Supplementary Fig.8: Expression of ATOH1 targets with NHE1 inhibition.

a, Dot plot showing the average expression level of ATOH1 targets in the secretory progenitor cluster. All dot plots are colored by average expression, 0 (population mean) ± SD. Data are analyzed by two-sided DECENT test. n.s., not statistically significant. *$p < 0.05$, **$p < 0.01$. $^{\textit{Creb3l1}\_\text{-EIPAvs+EIPA}}p = 0.9445$; $^{\textit{Creb3l1}\_\text{-Doxvs+Dox}}p = 0.001027$; $^{\textit{Kit}\_\text{-EIPAvs+EIPA}}p = 0.05513$; $^{\textit{Kit}\_\text{-Doxvs+Dox}}p = 0.4519$; $^{\textit{Cbfa2t3}\_\text{-EIPAvs+EIPA}}p = 0.1275$; $^{\textit{Cbfa2t3}\_\text{-Doxvs+Dox}}p = 0.2075$; $^{\textit{Sox9}\_\text{-EIPAvs+EIPA}}p = 0.05106$; $^{\textit{Sox9}\_\text{-Doxvs+Dox}}p = 0.4223$; $^{\textit{Sct}\_\text{-EIPAvs+EIPA}}p = 0.08146$; $^{\textit{Sct}\_\text{-Doxvs+Dox}}p = 0.7000$; $^{\textit{Hdac1}\_\text{-EIPAvs+EIPA}}p = 0.02023$; $^{\textit{Hdac1}\_\text{-Doxvs+Dox}}p = 0.06713$; $^{\textit{Gfi1}\_\text{-EIPAvs+EIPA}}p = 0.6872$; $^{\textit{Gfi1}\_\text{-Doxvs+Dox}}p = 0.01835$; $^{\textit{Maml3}\_\text{-EIPAvs+EIPA}}p = 0.1824$; $^{\textit{Maml3}\_\text{-Doxvs+Dox}}p = 0.9355$; $^{\textit{Dll4}\_\text{-EIPAvs+EIPA}}p = 0.1026$; $^{\textit{Dll4}\_\text{-Doxvs+Dox}}p = 0.5900$; $^{\textit{Cbfa2t2}\_\text{-EIPAvs+EIPA}}p = 0.6731$; $^{\textit{Cbfa2t2}\_\text{-Doxvs+Dox}}p = 0.1591$; $^{\textit{Neurog3}\_\text{-EIPAvs+EIPA}}p = 0.04360$; $^{\textit{Neurog3}\_\text{-Doxvs+Dox}}p = 0.9834$; **b,** CHGA immunolabeling as a marker for EEC of day 3 organoids maintained in the absence (Control) or presence of EIPA. Top panel representative images, bottom panel quantified data from 18 crypt regions in control organoids and 20 crypt regions with EIPA in separate preparations (n=2 biologically independent experiments, two-sided Welch's t-test, p = 1.284E-07) indicating significantly fewer EEC cells per crypt region determined by CD44 immunolabeling. Source data are provided as a Source Data file. Box plots are minimum to maximum, the box shows 25th-75th percentiles, and the central line is the median. Scale bar, 20 µm.

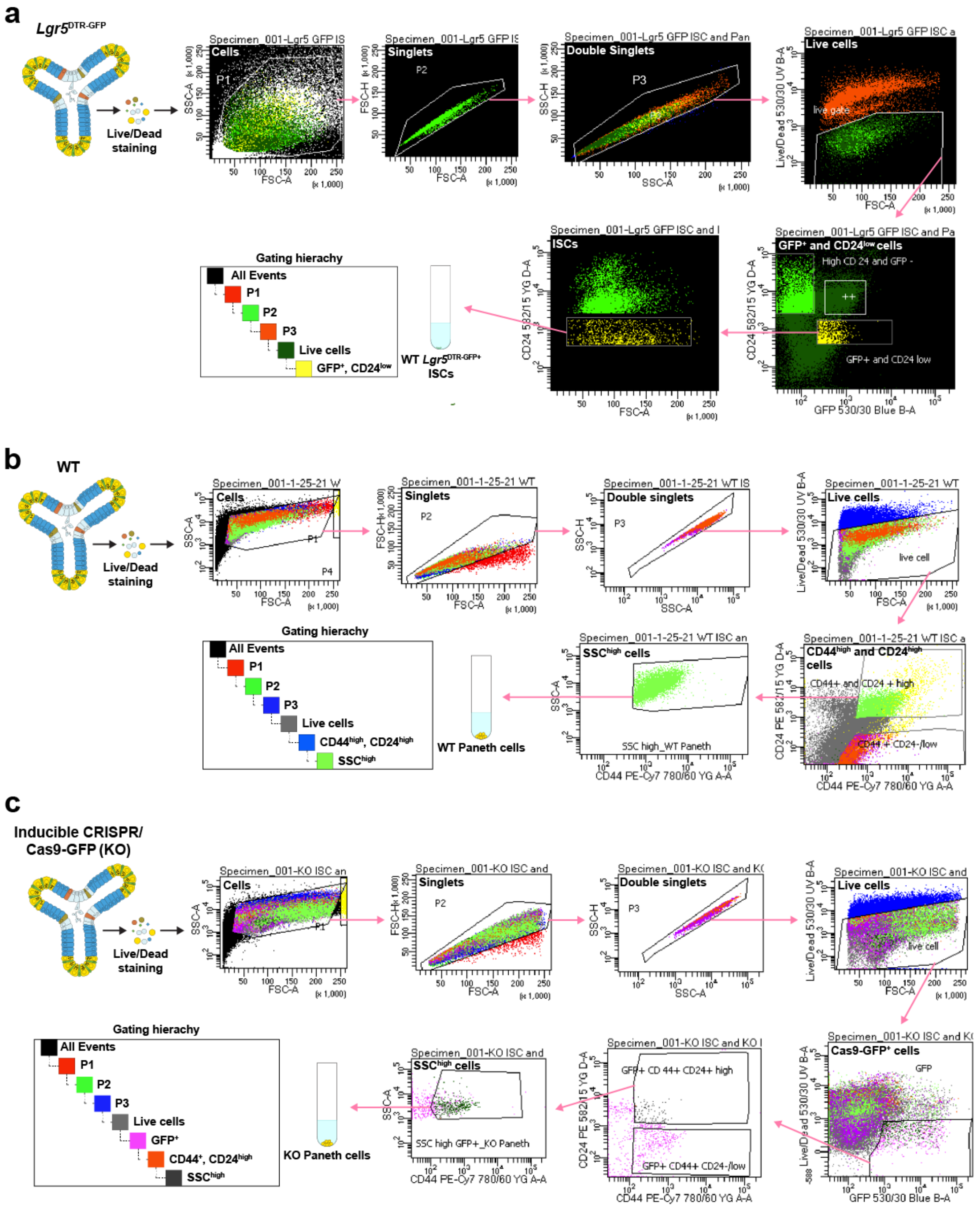

Supplementary Fig.9: FACS for isolating single ISCs and Paneth cells. a-c, The fluorescence-activated cell sorting (FACS) gating strategies for obtaining single WT ISCs, WT Paneth cells, and NHE1 KO Paneth cells. Organoids are dissociated into cell suspensions, followed by Live/Dead staining before sorting for desired cell types. WT single

ISCs (**a**) and Paneth cells (**b**) are isolated from the *Lgr5*$^{DTR\text{-}GFP}$ organoids and the WT organoids, respectively. In (c), single NHE1 KO Paneth cells are harvested from doxycycline (Dox)-inducible NHE1 CRISPR/Cas9-GFP organoids (+Dox).

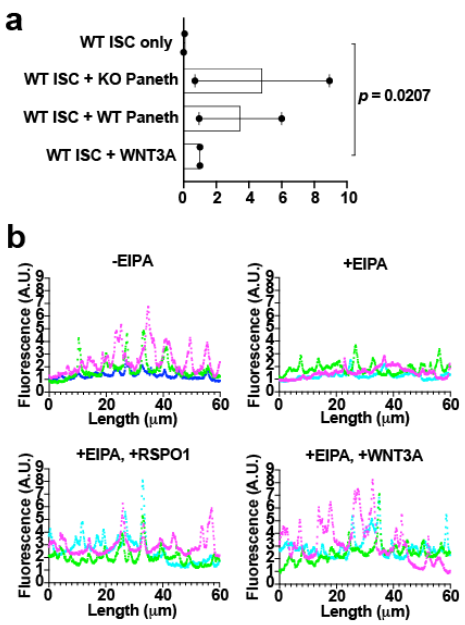

Supplementary Fig.10: Paneth cell function and EPHB2 staining.

a, Relative efficiency of *Lgr5*$^{+}$ ISC-Paneth cell single cell reassociation, determined by the number of organoids formed. Data are normalized to the positive control (single *Lgr5*$^{+}$ ISCs alone with WNT3A) and show the means of n=2 biologically independent experiments, with statistical analysis by the two-sided Wilcoxon test. Scatter dot plot, mean with range. Data with a statistically significant difference are specified with a *p* value, otherwise are not significantly different. Source data are provided as a Source Data file. **b,** Representative line plots of intensity of EPHB2 immunolabeling in crypt region of day 3 organoids with and without WNT rescue. Fluorescence intensity is determined by drawing a line across lateral layers of crypt cells using the line tool function in ImageJ. Each plot shows 3 individual crypt regions indicated by different colors (n=3 biologically independent experiments). Source data are provided as a Source Data file.

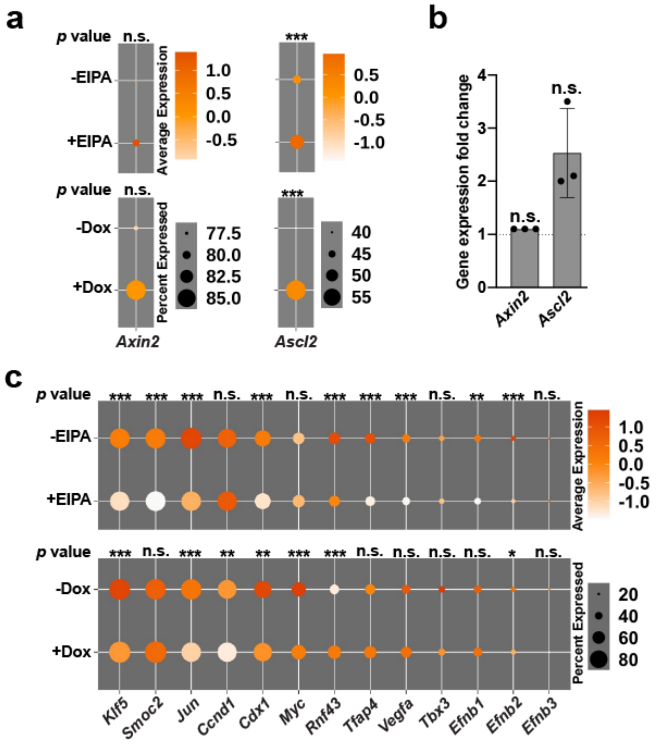

Supplementary Fig.11: Expression of WNT responsive genes with NHE1 inhibition.

a, Dot plots showing the average expression level of WNT target genes, *Axin2* and *Axcl2* in the *Lgr5*$^{+}$ ISC cluster. Dot plots are colored by average expression, 0 (population mean) ± SD. Statistical analysis was performed by two-sided DECENT test. *p* values in (**a**): $^{Axin2\_\text{-EIPAvs+EIPA}}p = 0.4031$; $^{Axin2\_\text{-Doxvs+Dox}}p = 0.6581$; $^{Ascl2\_\text{-EIPAvs+EIPA}}p = 7.82E\text{-}07$; $^{Ascl2\_\text{-Doxvs+Dox}}p = 6.29E\text{-}21$. **b,** Expression (mean ± SD) of *Axin2* and *Ascl2* in EIPA-treated organoids relative to non-treated organoids. Data are generated from n=3 biologically independent experiments, and analyzed by two-sided Wilcoxon Signed Rank Test. *p* values in (**b**): $^{Axin2}p = 0.25$; $^{Ascl2}p = 0.25$. Source data are provided as a Source Data file. **c,** Dot plot showing the average expression level of broad WNT targets in the *Lgr5*$^{+}$ ISC cluster. *Klf5* is a catenin-independent WNT responsive gene. Dot plots are colored by average expression, 0 (population mean) ± SD. Statistics was analyzed by two-sided DECENT test. *p* values in (**c**): $^{Klf5\_\text{-EIPAvs+EIPA}}p = 2.02E\text{-}38$; $^{Klf5\_\text{-Doxvs+Dox}}p = 6.44E\text{-}11$; $^{Smoc2\_\text{-EIPAvs+EIPA}}p = 8.76E\text{-}13$; $^{Smoc2\_\text{-Doxvs+Dox}}p = 0.9085$; $^{Jun\_\text{-EIPAvs+EIPA}}p = 1.43E\text{-}154$; $^{Jun\_\text{-Doxvs+Dox}}p = 1.20E\text{-}36$; $^{Ccnd1\_\text{-EIPAvs+EIPA}}p = 0.5286$; $^{Ccnd1\_\text{-Doxvs+Dox}}p = 0.002605$; $^{Cdx1\_\text{-EIPAvs+EIPA}}p = 1.28E\text{-}08$; $^{Cdx1\_\text{-Doxvs+Dox}}p = 0.001662$; $^{Myc\_\text{-EIPAvs+EIPA}}p = 0.5285$; $^{Myc\_\text{-Doxvs+Dox}}p = 0.0003073$; $^{Rnf43\_\text{-EIPAvs+EIPA}}p = 6.13E\text{-}06$; $^{Rnf43\_\text{-Doxvs+Dox}}p = 3.39E\text{-}11$; $^{Tfap4\_\text{-EIPAvs+EIPA}}p = 2.74E\text{-}08$; $^{Tfap4\_\text{-Doxvs+Dox}}p = 0.7542$; $^{Vegfa\_\text{-EIPAvs+EIPA}}p = 0.0004146$; $^{Vegfa\_\text{-Doxvs+Dox}}p = 0.3233$; $^{Tbx3\_\text{-EIPAvs+EIPA}}p = 0.3609$; $^{Tbx3\_\text{-Doxvs+Dox}}p = 0.1180$; $^{Efnb1\_\text{-EIPAvs+EIPA}}p = 0.001588$; $^{Efnb1\_\text{-Doxvs+Dox}}p = 0.8447$; $^{Efnb2\_\text{-EIPAvs+EIPA}}p = 4.20E\text{-}09$; $^{Efnb2\_\text{-Doxvs+Dox}}p = 0.01375$; $^{Efnb3\_\text{-EIPAvs+EIPA}}p = 0.3423$; $^{Efnb3\_\text{-Doxvs+Dox}}p = 0.2812$. All plots: $*p < 0.05$, $**p < 0.01$, $***p < 0.001$. n.s., not statistically significant.
