# Supplementary material for: Intracellular pH dynamics regulates intestinal stem cell lineage specification
Source: Nat Commun. 2023 Jun 23;14:3745. doi: 10.1038/s41467-023-39312-9 (PMC10290085; doi:10.1038/s41467-023-39312-9)
Supplement: Supplementary file 3 — Description of Additional Supplementary Files [file 41467_2023_39312_MOESM3_ESM.pdf]

Description of Additional Supplementary Files

Supplementary Movie 1. Video of mCherry-SEpHluroin fluorescence ratio in a budding crypt (day 1 to day 2), corresponding to Fig.1d. Fluorescence ratios are calibrated to pH values using nigericin-containing buffers to show that a dynamic pHi gradient, lower at the crypt base and higher at the crypt neck, is generated during crypt budding. Left view, bright field. Right view, mCherry/SEpHluorin ratio.

Supplementary Movie 2. Video of crypt budding in an untreated control *Lgr5*$^{DTR\text{-}GFP}$ organoid from day 0 to day 3, corresponding to Fig.2a. The crypt region elongates and forms a budded crypt. Left view, bright field. Right view, *Lgr5*$^{+}$ ISC.

Supplementary Movie 3. Video of impaired crypt budding in an EIPA-treated *Lgr5*$^{DTR\text{-}GFP}$ organoid from day 0 to day 3, corresponding to Fig.2a. The crypt region initiates a protrusion but fails to form a budded crypt. Left view, bright field. Right view, *Lgr5*$^{+}$ ISC.

Supplementary Movie 4. Video shows the production of secretory cells in the crypt region of an untreated control *Atoh1*^{CreERT2};*Rosa26*^{tdTomato} organoid from day 2 to day 3, corresponding to Fig.5f. Newly produced ATOH1^{+} (tdTomato^{+}) secretory cells appear in the crypt as the crypt elongates and forms a bud.

Supplementary Movie 5. Video shows attenuated production of secretory cells in the crypt region of an EIPA-treated *Atoh1*^{CreERT2};*Rosa26*^{tdTomato} organoid from day 2 to day 3, corresponding to Fig.5f. No evident increase of newly produced ATOH1^{+}(tdTomato^{+}) secretory cells appears in the unbudded crypt region
