# Supplementary material for: Intracellular pH dynamics regulates intestinal stem cell lineage specification
Source: Nat Commun. 2023 Jun 23;14:3745. doi: 10.1038/s41467-023-39312-9 (PMC10290085; doi:10.1038/s41467-023-39312-9)
Supplement: Supplementary file 9 — Reporting Summary [file 41467_2023_39312_MOESM9_ESM.pdf]

nature portfolio

Corresponding author(s): Todd Nystul
Diane Barber

Last updated by author(s): May 26, 2023

Reporting Summary

Nature Portfolio wishes to improve the reproducibility of the work that we publish. This form provides structure for consistency and transparency in reporting. For further information on Nature Portfolio policies, see our Editorial Policies and the Editorial Policy Checklist.

Statistics

For all statistical analyses, confirm that the following items are present in the figure legend, table legend, main text, or Methods section.

n/a	Confirmed	
[ ]	[x]	The exact sample size (*n*) for each experimental group/condition, given as a discrete number and unit of measurement
[ ]	[x]	A statement on whether measurements were taken from distinct samples or whether the same sample was measured repeatedly
[ ]	[x]	The statistical test(s) used AND whether they are one- or two-sided<br>*Only common tests should be described solely by name; describe more complex techniques in the Methods section.*
[x]	[ ]	A description of all covariates tested
[x]	[ ]	A description of any assumptions or corrections, such as tests of normality and adjustment for multiple comparisons
[ ]	[x]	A full description of the statistical parameters including central tendency (e.g. means) or other basic estimates (e.g. regression coefficient) AND variation (e.g. standard deviation) or associated estimates of uncertainty (e.g. confidence intervals)
[ ]	[x]	For null hypothesis testing, the test statistic (e.g. *F*, *t*, *r*) with confidence intervals, effect sizes, degrees of freedom and *P* value noted<br>*Give P values as exact values whenever suitable.*
[x]	[ ]	For Bayesian analysis, information on the choice of priors and Markov chain Monte Carlo settings
[x]	[ ]	For hierarchical and complex designs, identification of the appropriate level for tests and full reporting of outcomes
[ ]	[x]	Estimates of effect sizes (e.g. Cohen's *d*, Pearson's *r*), indicating how they were calculated

Our web collection on statistics for biologists contains articles on many of the points above.

Software and code

Policy information about availability of computer code

Data collection	Imaging was performed on a Nikon Ti-E microscope equipped with a live-cell imaging chamber maintained with 5% CO2 at 37°C, a 40X water objective, 488 nm, 560 nm, and 590 nm excitation lasers. and a Photometrics cMYO cooled CCD camera. FACS was performed on the BD FACSAria instrument (FACSDiva 8.0.1).
Data analysis	The raw sequence outputs were filtered and aligned (mouse reference, mm10) to produce feature-barcode matrices using the Cell Ranger pipeline 6.1.2 on 10X Genomics Cloud Analysis. Single-cell feature-barcode matrices were analyzed using Seurat 4.1 and related packages on RStudio server 2022.01.999. Full custom code will be made available at https://zenodo.org/record/7922816 .The statistical analysis was performed using GraphPad Prism 8 and RStudio.

For manuscripts utilizing custom algorithms or software that are central to the research but not yet described in published literature, software must be made available to editors and reviewers. We strongly encourage code deposition in a community repository (e.g. GitHub). See the Nature Portfolio guidelines for submitting code & software for further information.

Data

Policy information about availability of data

All manuscripts must include a data availability statement. This statement should provide the following information, where applicable:

- Accession codes, unique identifiers, or web links for publicly available datasets
- A description of any restrictions on data availability
- For clinical datasets or third party data, please ensure that the statement adheres to our policy

Source data are provided with this paper. The single-cell RNA sequencing data generated in this study have been deposited in NCBI gene expression omnibus under accession code GSE211097 [https://www.ncbi.nlm.nih.gov/geo/query/acc.cgi?acc=GSE211097].

Code for bioinformatic analysis is deposited in a GitHub repository and is freely accessible [https://zenodo.org/record/7922816].

Human research participants

Policy information about studies involving human research participants and Sex and Gender in Research.

Reporting on sex and gender	NA
Population characteristics	NA
Recruitment	NA
Ethics oversight	NA

Note that full information on the approval of the study protocol must also be provided in the manuscript.

Field-specific reporting

Please select the one below that is the best fit for your research. If you are not sure, read the appropriate sections before making your selection.

- [x] Life sciences
- [ ] Behavioural & social sciences
- [ ] Ecological, evolutionary & environmental sciences

For a reference copy of the document with all sections, see nature.com/documents/nr-reporting-summary-flat.pdf

Life sciences study design

All studies must disclose on these points even when the disclosure is negative.

Sample size	No sample size calculation was performed. In general, for each experiment, sampling was performed with at least 80% of all available organoids in the culture.
Data exclusions	No data exclusion
Replication	Experimental finding were generally replicated with at least 3 independent biological repeats. Replication was successful.
Randomization	The organoid culture used in each experiment intrinsically consists of ten to hundreds individual organoids. More than 80% of healty WT organoids, drug-treated organoids, or engineered organoids that match a perticular requirement of genetic selection (e.g fluorescent) were randomly selected.
Blinding	Not possible. Experiment design, preparation, data collection, and data analysis were carried out by the same person. Partial blinding was involved in the data analysis.

Reporting for specific materials, systems and methods

We require information from authors about some types of materials, experimental systems and methods used in many studies. Here, indicate whether each material, system or method listed is relevant to your study. If you are not sure if a list item applies to your research, read the appropriate section before selecting a response.

Materials & experimental systems

n/a	Involved in the study
[ ]	[x] Antibodies
[x]	[ ] Eukaryotic cell lines
[x]	[ ] Palaeontology and archaeology
[ ]	[x] Animals and other organisms
[x]	[ ] Clinical data
[x]	[ ] Dual use research of concern

Methods

n/a	Involved in the study
[x]	[ ] ChIP-seq
[ ]	[x] Flow cytometry
[x]	[ ] MRI-based neuroimaging

Antibodies

Antibodies used

Anti-CD44, BioLegend, Catalog# 103030
Anti-CD24, BD, Catalog#553262
Anti-lysozyme, Dako, Catalog# EC 3.2.1.17
Anti-EPHB2, R&D, Catalog# AF496
Anti-DLL1, R&D, Catalog# AF5026
Anti-Aldolase B, Abcam, Catalog# ab75751
Anti-Ki67, (gift)
Anti-Chromogranin A, (gift)

Validation

Anti-CD44, BioLegend, Catalog# 103030
Isotype Control:
PE/Cyanine7 Rat IgG2b, κ Isotype Ctrl
Verified Reactivity:
Mouse, Human
Reported Reactivity::
Chimpanzee, Baboon, Cynomolgus, Rhesus, Squirrel Monkey, Horse, Cow, Pig, Dog, Cat
Antibody Type:
Monoclonal
Host Species:
Rat
Immunogen:
Dexamethasone-induced myeloid leukemia M1 cells

Anti-CD24, BD, Catalog#553262
Reactivity:
Mouse
Isotype:
Rat DA, also known as DA/HA IgG2b, κ
Immunogen:
C57BL/10 Mouse Splenic T Lymphocytes
Application:
Flow cytometry (Routinely Tested)
Concentration:
0.2 mg/ml

Anti-lysozyme, Dako, Catalog# EC 3.2.1.17
Clone:
Polyclonal
Immunogen:
Lysozyme isolated from urine of patients with monocytic leukaemia.
Species:
Rabbit Anti-Human

Anti-EPHB2, R&D, Catalog# AF467
Species Reactivity:
Human, Mouse
Specificity:
Detects mouse and human EphB2 in direct ELISAs and Western blots. In Western blots, approximately 5% cross-reactivity with recombinant rat (rr) EphB1, recombinant mouse (rm) EphA8, rmEphA6, rmEphB6, rmEphA3, rmEphA4, rmEphA7, rrEphA5, recombinant human EphA1, rmEphA2 and rmEphB3 is observed.
Source:
Polyclonal Goat IgG
Purification:

Antigen Affinity-purified
Immunogen:
Mouse myeloma cell line NS0-derived recombinant mouse EphB2
Val27-Lys548
Accession # P54763

Anti-DLL1, R&D, Catalog# AF5026
Species Reactivity:
Mouse, Rat
Specificity:
Detects mouse and rat DLL1 in direct ELISAs and Western blots. In direct ELISAs, approximately 40% cross-reactivity with recombinant human (rh) DLL1 and less than 1% cross-reactivity with rhDLL3, rhDLL4, and recombinant mouse DLL4 is observed.
Source:
Polyclonal Sheep IgG
Purification:
Antigen Affinity-purified
Immunogen:
Mouse myeloma cell line NS0-derived recombinant mouse DLL1
Ser22-Gln516
Accession # Q61483

Anti-Aldolase B, Abcam, Catalog# ab75751
Description:
Rabbit monoclonal [EPR3138Y] to Aldolase B + Aldolase C
Host species:
Rabbit
Tested applications:
Suitable for: Flow Cyt, WB, IHC-Pmore details
Species reactivity:
Reacts with: Mouse, Rat, Human
Immunogen:
Synthetic peptide within Human Aldolase B aa 250-350. The exact sequence is proprietary.
Database link: P05062

Animals and other research organisms

Policy information about studies involving animals; ARRIVE guidelines recommended for reporting animal research, and Sex and Gender in Research

Laboratory animals	The C57BL/6J (male, 10 weeks old) and mTmG (female, 10 weeks old) adult mice were used for isolating fresh crypts for ex vivo pHi measurement.
Wild animals	No wild animal was involved in this study.
Reporting on sex	Not indicated, as our findings were not sex related.
Field-collected samples	Field-collected samples were not involved in this study.
Ethics oversight	Animal-use protocols were approved by the Institutional Animal Care and Use Committee (IACUC) of the University of California at San Francisco (AN197697-00 and AN192878-01G), and all experiments were conducted in accordance with the specified guidelines.

Note that full information on the approval of the study protocol must also be provided in the manuscript.

Flow Cytometry

Plots

Confirm that:

- [x] The axis labels state the marker and fluorochrome used (e.g. CD4-FITC).
- [x] The axis scales are clearly visible. Include numbers along axes only for bottom left plot of group (a 'group' is an analysis of identical markers).
- [x] All plots are contour plots with outliers or pseudocolor plots.
- [x] A numerical value for number of cells or percentage (with statistics) is provided.

Methodology

Sample preparation	To obtain single-cell suspension from the organoids without or with NHE1 inhibition (-/+ EIPA, -/+ Dox), organoid cultures were first washed with the cold (4°C) advanced DMEM/F12 (Invitrogen, 12634-028) supplemented with 10 mM HEPES (Invitrogen, 15630-080), 1 mM N-acetylcysteine (Sigma-Aldrich, A7250), 1X glutaMAX (Invitrogen, 35050-061), and 1X pen/strep. After the washing, wells containing the organoids were incubated with 300 uL cold (4°C) Corning recovery solution (Corning, 354253) for 15 min to melt the Matrigel. Next, organoids were mechanically disrupted with the P1000 pipette tips first and then trypsinized with RT TrypLE Express (Gibco, 1952062) for up to 15 min to disassociate into single cells. Single cells were then suspended within a sorting buffer that contained Hanks' balanced salt solution (HBSS) with 3% m/v FBS, 10 mM HEPES and 5 mM EDTA. After staining with Live/Dead (Invitrogen, L23105, 1:1000) in the sorting buffer, fluorescence-activated cell sorting (FACS) was performed.
Instrument	BD FACSAira instrument
Software	FACSDiva 8.0.1
Cell population abundance	The abundance of cell population within post-FACS was provided in the methods. Purity was more than 90% and determined by flow cytometry using the Live/Dead (Invitrogen, L23105, 1:1000) stain.
Gating strategy	For scRNA-seq: live cells (-/+ EIPA and -Dox conditions) or live GFP+ cells (+Dox condition) are enriched.<br>For ISC-Paneth cell reassociation assay: Live Lgr5+ ISCs (Lgr5-DTR-GFPhigh, CD44high, CD24low, and side-scatterlow), WT Paneth cells (Lgr5-DTR-GFPnegative, CD44high, CD24high, and side-scatterhigh), and NHE1-silenced Paneth cells (Cas9-GFPhigh, CD44high, CD24high, and side-scatterhigh) were sorted respectively.

☒ Tick this box to confirm that a figure exemplifying the gating strategy is provided in the Supplementary Information.
